# Hepatocellular carcinoma patients may benefit from left hemi-hepatectomy with caudate lobectomy

**DOI:** 10.12669/pjms.291.3216

**Published:** 2013

**Authors:** Hongyu Li, Bo Li, Yonggang Wei

**Affiliations:** 1 Hongyu Li, Department of Liver and Vascular Surgery, Center of Liver Transplantation, West China Hospital, Sichuan University, Chengdu 610041, Sichuan Province, China.; 2 Bo Li, PhD, MD, Department of Liver and Vascular Surgery, Center of Liver Transplantation, West China Hospital, Sichuan University, Chengdu 610041, Sichuan Province, China.; 3 Yonggang Wei, MD, Department of Liver and Vascular Surgery, Center of Liver Transplantation, West China Hospital, Sichuan University, Chengdu 610041, Sichuan Province, China.

 In order to archive a better prognosis, left hemi-hepatectomy is usually used as the first-line treatment for the hepatocellular carcinoma (HCC) patients with left portal vein tumor thrombus. Because of the short distance between hilar bile duct and caudate lobe bile duct, hilar cholangiocarcinoma has a high metastasis rate on caudate lobe ducts. Caudate lobectomy contributes to improvement of disease-free survival (DFS) and overall survival (OS) in type III hilar cholangiocarcinoma.^1^^,^^2^ The left portal vein is closer to the caudate lobe portal vein in structure, and as a result of these, we hypothesis that left hemi-hepatectomy with caudate lobectomy may benefit HCC patients which have left portal vein tumor thrombus involved.

 Thirty HCC adult patients with left portal vein tumor thrombus were admitted in our medical center from January 2008 to September 2009. All of the patients were Child-Pugh A cirrhosis with preserved liver function and normal ICG-15 clearance rate preoperatively and, then, diagnosed with HCC according to postoperative pathologic results. Left hemi-hepatectomy was performed for all patients, 10 with additional caudate lobectomy (Group A) and 20 without (Group B). The demographics and symptoms of presentation were comparable. The median postoperative bilirubin level and hospital stays between two groups were not significantly different. There were no mobility and mortality in perioperative period. Patients had a significantly higher 1-, and 3-year OS rates in Group A, compared to Group B, (43% vs 32%, P<0.001; 16% vs 11%, P=0.013) and higher 1-, and 3-year DFS rates (15% vs 9.6%, P<0.001; 8% vs 4.8%, P<0.001). Multivariate analysis for OS and DFS rates found that caudate lobectomy (p = 0.020) and poorly differentiated HCC (p = 0.001) were positive and negative prognostic factors, respectively.

 As a result of the shorter distance between left portal vein and caudate lobe portal vein, local invasion and metastasis may cause potential vascular invasion in caudate lobe in HCC patients with left portal vein tumor thrombus. Our initial study proved that left hepatetomy with caudate lobectomy might have better OS and DFS rates and did not enhance the postoperative liver dysfunction risk. We consider that HCC patients with left portal vein tumor thrombus may benefit from such surgical approach.

## Notes

***Abbreviation:*** HCC: hepatocellular carcinoma, OS: overall survival, DFS: disease-free survival.
